# Prognostic value of mitotic checkpoint protein BUB3, cyclin B1, and pituitary tumor-transforming 1 expression in prostate cancer

**DOI:** 10.1038/s41379-019-0418-2

**Published:** 2019-12-04

**Authors:** Elin Ersvær, Wanja Kildal, Ljiljana Vlatkovic, Karolina Cyll, Manohar Pradhan, Andreas Kleppe, Tarjei S. Hveem, Hanne A. Askautrud, Marco Novelli, Håkon Wæhre, Knut Liestøl, Håvard E. Danielsen

**Affiliations:** 1 Institute for Cancer Genetics and Informatics, Oslo University Hospital, NO-0424 Oslo, Norway; 2 Department of Pathology, Oslo University Hospital, NO-0424 Oslo, Norway; 3 Department of Informatics, University of Oslo, NO-0316 Oslo, Norway; 4 Research Department of Pathology, University College London Medical School, London, WX1E 6DE UK; 5 Nuffield Division of Clinical Laboratory Sciences, University of Oxford, Oxford, OX3 9DU UK

**Keywords:** Prostate cancer, Tumour biomarkers, Tumour heterogeneity

## Abstract

The mitotic checkpoint protein BUB3, cyclin B1 (CCNB1) and pituitary tumor-transforming 1 (PTTG1) regulates cell division, and are sparsely studied in prostate cancer. Deregulation of these genes can lead to genomic instability, a characteristic of more aggressive tumors. We aimed to determine the expression levels of BUB3, CCNB1, and PTTG1 as potential prognostic markers of recurrence after radical prostatectomy. Protein levels were determined by immunohistochemistry on three formalin-fixed paraffin-embedded tissue sections from each of the 253 patients treated with radical prostatectomy. Immunohistochemistry scores were obtained by automated image analysis for CCNB1 and PTTG1. Recurrence, defined as locoregional recurrence, distant metastasis or death from prostate cancer, was used as endpoint for survival analysis. Tumors having both positive and negative tumor areas for cytoplasmic BUB3 (30%), CCNB1 (28%), or PTTG1 (35%) were considered heterogeneous. Patients with ≥1 positive tumor area had significantly increased risk of disease recurrence in univariable analysis compared with patients where all tumor areas were negative for cytoplasmic BUB3 (hazard ratio [HR] = 2.18, 95% confidence interval [CI] 1.41–3.36), CCNB1 (HR = 2.98, 95% CI 1.93–4.61) and PTTG1 (HR = 1.91, 95% CI 1.23–2.97). Combining the scores of cytoplasmic BUB3 and CCNB1 improved risk stratification when integrated with the Cancer of the Prostate Risk Assessment post-Surgical (CAPRA-S) score (difference in concordance index = 0.024, 95% CI 0.001–0.05). In analysis of multiple tumor areas, prognostic value was observed for cytoplasmic BUB3, CCNB1, and PTTG1.

## Introduction

Intra-tumor heterogeneity is common in prostate cancer, and may to some extent explain the difficulties in establishing useful molecular markers for this disease [1]. Intra-tumor heterogeneity is a beneficial trait for cancer progression and it likely develops due to genomic instability [2], which can be induced by defects in the mechanisms that regulate mitosis. The mitotic checkpoint controls mitosis through the mitotic checkpoint complex, where the mitotic checkpoint protein BUB3 is a key component [3]. Correct attachment of the kinetochores inactivates the mitotic checkpoint complex, which facilitates the degradation of cyclin B1 (CCNB1) and pituitary tumor-transforming 1 (PTTG1), and determines exit from mitosis [3].

Defects in the mitotic checkpoint may lead to chromosome mis-segregation, generating aneuploidy which is a marker of poor prognosis in many cancer types [4, 5]. The impairment in function of the mitotic checkpoint is often associated with changes in the levels of proteins involved in the checkpoint [4]. The expression of BUB3, CCNB1, and PTTG1 has been shown to correlate with tumor grade and prognosis in some cancers [6–8]. However, their prognostic role in prostate cancer is unclear.

As better risk stratification of patients with prostate cancer is needed [9], we aimed to investigate whether the expression of BUB3, CCNB1 and PTTG1 could independently predict recurrence after radical prostatectomy. Protein levels were assessed by immunohistochemistry and performed on three tumor-containing tissue sections for each patient (*n* = 253) in order to account for intra-tumor heterogeneity. The mRNA counts were determined in one tumor-containing tissue sample for each patient.

## Materials and methods

### Patients and specimens

**Fig. 1:**
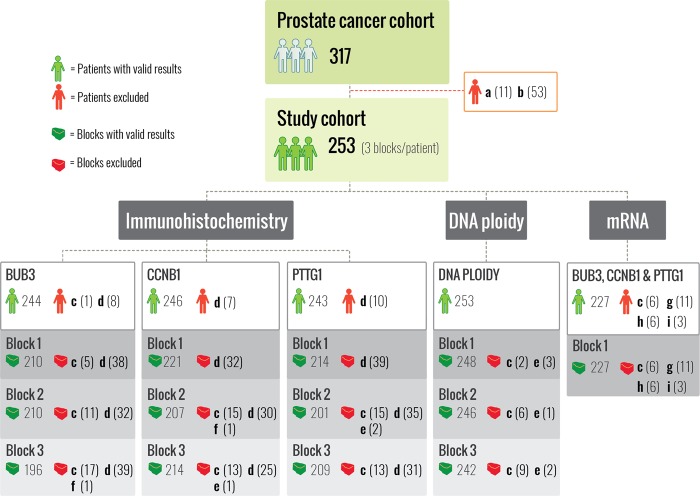
Overview of patients and methods. Immunohistochemical analyses of BUB3, cyclin B1 (CCNB1) and pituitary tumor-transforming 1 (PTTG1) and DNA ploidy analyses were performed on tissue sections cut from three separate tumor containing blocks. We included tumor areas ≥4 mm^2^, as measured on the diagnostic hematoxylin and eosin tissue sections. Some of the tissue blocks had been sectioned for other studies before they were included in this study. Consequently, some tumor areas were reduced to <4 mm^2^ and 53 patients were excluded due to insufficient material fulfilling the inclusion criteria. The mRNA counts were analyzed with the NanoString technology in one sample corresponding to block 1 used in the immunohistochemistry study. The green patients/blocks represent the number of patients/samples with valid results, whereas the red ones represent excluded patients/samples. Exclusion criteria: ^a^Patients missing clinical data. ^b^Not fulfilling the selection criteria: three tumor containing tissue blocks were selected based on highest Gleason score and/or previously measured worst DNA ploidy. ^c^No tumor in immunostained section or hematoxylin and eosin control section for DNA ploidy. ^d^≥95% of tumor area fell off during the immunohistochemistry procedure. ^e^Other technical issues. ^f^Missing. ^g^Reduced Quality Control parameters detected by nSolver. ^h^>40% infiltrating benign glands. ^i^<50 ng mRNA.

A cohort of 317 patients with primary prostate cancer was treated with radical prostatectomy at a tertiary comprehensive cancer center in Norway between 1987 and 2005 (The Norwegian Radium Hospital, Oslo). The basis of prostatectomy was preoperative absence of known metastases, age <75 years and life expectancy of ≥10 years. Neoadjuvant therapy was not given to any of the patients included in the analyses. Radiation and androgen deprivation were only given after indication of recurrence. This study adhered to the REporting recommendations for tumor MARKer prognostic studies reporting criteria [10] and was approved by the Regional Committees for Medical and Health Research Ethics (REK), Norway (REK no. S-07443a). All tissue sections with tumors were Gleason graded by an experienced uropathologist (LV) according to the updated 2005 International Society of Urological Pathology Consensus guidelines [11]. Three tumor-containing tissue blocks were selected based on the highest Gleason score and/or previously reported worst DNA ploidy status [1]. Sufficient tumor material for analyses was available for 253 patients (Fig. 1). Tumor areas that measured <4 mm^2^ on a diagnostic hematoxylin and eosin tissue section or immune stained sections were excluded. Tumor areas within one section were analyzed separately if they were situated ≥3 mm apart. Two to four tumor areas were found on 104 of the 759 included blocks.

### Immunohistochemistry

Three micrometers thick tissue sections were cut and mounted on Superfrost plus slides (Thermo Scientific, Waltham, MA), dried at 60 °C and stored at −80 °C. The Envison FLEX + system/Dako Autostainer Link 48 (Agilent Technologies, Santa Clara, CA) was used for the immunohistochemistry procedure. Parallel tissue sections were incubated with antibodies against BUB3, CCNB1, and PTTG1 (Supplementary Table 1). The specificity of the BUB3 antibody was confirmed with the Human Bub3 peptide (Abcam, Cambridge, UK), following the manufacturer’s recommendations. Positive and negative controls were included in each run. All sections were counterstained with hematoxylin and scanned at ×40 (NanoZoomer HT, Hamamatsu Photonics, Hamamatsu, Japan). Tumor areas with ≥95% of the tumor tissue lost during processing were excluded (301 of 2277 tissue sections, 13%, Fig. 1).

### Visual scoring of BUB3

The complete tumor areas were evaluated using NDP.view2 (Hamamatsu Photonics, Hamamatsu, Japan) at ×10 magnification (EE). Pre-defined thresholds were used to categorize the scores. As there is no consensus on how to score BUB3, the thresholds were selected after a visual examination of the staining pattern of BUB3. Nuclear BUB3 was normally expressed in all tumor cells and a tumor area was considered positive when ≥99% of the tumor cells had nuclear BUB3. Cytoplasmic expression was observed in single cells scattered throughout the tumor area, and were commonly observed in mitotic cells. A cut-off value of >5% was chosen so that a sample was only considered positive when the levels of cytoplasmic BUB3 exceeded the background levels of positivity. Cytoplasmic BUB3 was also scored independently in 1/3 of the tissue sections by a pathologist (MP). BUB3 could not be scored automatically due to overlapping color spectra between positive cytoplasm and nuclei. In addition, a less accurate count was obtained for poorly differentiated tumors, as the accuracy of the count was dependent on the degree of clustering of cells.

### Automated scoring of CCNB1 and PTTG1

The complete tumor areas were marked manually in ImmunoPath (Room4 Group Ltd, Crowborough, UK), while avoiding artifacts, lymphocytes, and intermixed benign glands. Tiles that measured 500 µm × 500 µm were generated within the annotated areas. Separate image analysis protocols were developed using images, which represented the staining variation for each protein. The nuclei were detected using a Count Transform, and the optimal color ranges for the positive nuclei were specified based on hue, saturation, and value thresholds. Two different commands were used to enhance the signal to noise in the marked area. Holes in the segmentation of a cell may occur due to color variation in the immune stain of the cell, and could exclude a positive cell if the segmented area was too small. A more accurate score was achieved using the “hole fill” function, which adds segmentation pixels to the hole. The “median 3 × 3” function was used to replace each segmented pixel with the median of the surrounding segmented pixels in a 3 by 3 window. This function removes small and unspecific segmentations and fills in missing pixels in a segmented object. Furthermore, segmented objects that did not represent positively stained cells were excluded by removing both small (<100 pixels) and large (>2000 pixels) objects (Supplementary Table 2). The number of positive cells and the size of the tumor area were reported by ImmunoPath and positive cells/mm^2^ was calculated.

### DNA ploidy and RNA analyses

DNA ploidy was analyzed by image cytometry as previously described [1, 5]. The samples were classified as diploid or non-diploid (tetraploid and aneuploid). Quantification of RNA was performed on 297 tumor areas from one tissue block from the 253 patients as described in supplementary methods.

### Thresholds

In the subsequent analyses, the automatic scores of CCNB1 and PTTG1 were dichotomized by the 75th percentile as described in Supplementary Fig. 1, whereas the mRNA counts of BUB3 were dichotomized by the 25th percentile as described in Supplementary Fig. 2.

### Statistical analysis

Time to recurrence, defined in accordance with Punt and co-workers [12], was the primary endpoint and was calculated from surgery to recurrence of disease or 31.12.2008. Recurrence rates were compared with the SPSS software (v23.0, IBM Corporation, Armonk, NY) using the Mantel–Cox log-rank test in multivariable analysis of categorical variables and the Wald chi-squared test in univariable analysis of continuous variables and in multivariable analyses. Concordance index (c-index) [13] with bias-corrected and accelerated confidence interval (CI) over 10 000 bootstraps [14] was computed in Stata/SE 15.1 (StataCorp, College Station, TX). Two-sided *p* value for test of difference in c-index was calculated as 1 minus the confidence level of the largest bias-corrected and accelerated CI that did not contain 0. Two-sided *p* values <0.05 were considered statistical significant.

## Results

### Patients

The patients were followed as described by Wæhre et al. [15] for a median of 11.1 (interquartile range (IQR) 7.5–14.2) years and recurrence was observed in 86/253 (34%) patients. Local recurrence was most frequently confirmed by biopsy, alternatively by palpation and/or ultrasound, whereas metastases were confirmed by scintigraphy. Local recurrence was observed in 36 patients, metastases were confirmed in 22 patients, and 23 patients had both local recurrence and metastases. The remaining five patients died of prostate cancer according to their death certificate. An overview of clinicopathological data and included samples are presented in Table 1 and Fig. 1.

**Table 1:** Clinicopathological data.

Variable	*n*	No recurrence, *n*	Recurrence, *n*	*p* value^a^
Study Cohort	253	167	86	
Age at surgery				0.642
Median (IQR)	62 (58–67)	62 (58–67)	63 (58–67)	
Age at surgery				0.769
≤65	162	108	54	
>65	91	59	32	
Preoperative PSA (ng/ml)				**<0.001**
≤6	57	49	8	
>6 and ≤10	48	41	7	
>10 and ≤20	84	48	36	
>20	62	29	33	
Missing	2	0	2	
Gleason score				**<0.001**
≤6	11	11	0	
3 + 4	92	83	9	
4 + 3	77	48	29	
≥8	73	25	48	
Surgical margins				**0.001**
Negative	91	72	19	
Positive	162	95	67	
Extracapsular extension				**<0.001**
Absent	55	50	5	
Present	196	115	81	
Missing	2	2	0	
Seminal vesicle invasion				**<0.001**
Absent	187	144	43	
Present	66	23	43	
Lymph node invasion				**0.014**
Absent	239	162	77	
Present	14	5	9	
CAPRA-S risk group				**<0.001**
Low (0–2)	30	30	0	
Intermediate (3–5)	87	75	12	
High (6–12)	132	60	72	
Missing	4	2	2	
DNA ploidy				**<0.001**
Diploid	142	109	33	
Tetraploid	67	34	33	
Aneuploid	44	24	20	
Follow-up, years				**0.047**
Median (IQR)	11.1 (7.5–14.2)	10.1 (7.3–14.1)	12.3 (9.1–14.3)	

*IQR* inter quartile range

^a^Associations were evaluated using the Pearson’s χ^2^ test for categorical variables, Kendall’s τ test for ordinal variables and Mann–Whitney’s *U*-test for continuous variables

### Protein

**Fig. 2:**
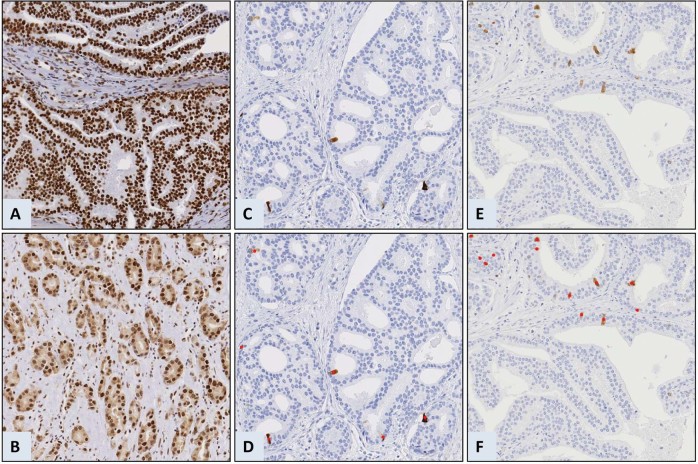
Examples of protein labeling and scoring in tissue samples from prostate cancer. Nuclear and cytoplasmic protein levels of mitotic checkpoint protein BUB3, cyclin B1 (CCNB1) and pituitary tumor-transforming 1 (PTTG1) was scored by assessing the whole tumor area. The tiles represent examples of (**a**) nuclear BUB3, (**b**) cytoplasmic and nuclear BUB3, (**c**) CCNB1, and (**e**) PTTG1 protein levels. Cells identified by ImmunoPath as positive are marked in red for (**d**) CCNB1 and (**f**) PTTG1. Negative cells are apparent by the blue counterstain of the nuclei. Each tile is 500 µm × 500 µm.

BUB3 (*n* = 244,616 samples) was expressed in the nuclei of both benign epithelial and tumor cells. Additional cytoplasmic stain was observed in a subset of the tumor cells (Fig. 2). The inter-observer agreement of the cytoplasmic BUB3 score was substantial (Cohen’s κ = 0.71, 92% with equal score). We observed 78 (32%) patients with ≥1 cytoplasmic BUB3 positive tumor area and 126 (52%) patients with ≥1 tumor area with decreased levels of nuclear BUB3. Cytoplasmic and nuclear stain of CCNB1 (*n* = 246,642 samples) and PTTG1 (*n* = 243,624 samples) were observed in a low fraction of the tumor cells (Fig. 2). The median positive cells/mm^2^ was 31 (IQR 18–55) for CCNB1 and 24 (IQR 14–40) for PTTG1 (Supplementary Fig. 1). Positive scores in ≥1 tumor area were observed for 94 (38%) patients for CCNB1 and 103 (42%) patients for PTTG1. The categorized scores of CCNB1 and PTTG1 (n = 238) were significantly correlated (Pearson’s *r* = 0.60, *p* < 0.001, 81% with equivalent scores).

### mRNA

We observed a median of 67 (IQR 52–81) BUB3 transcripts and decreased counts of BUB3 mRNA in 56 patients (*n* = 227, Fig. 1). The median expression was 11 (IQR 7–15) mRNA transcripts for CCNB1 and 4 (IQR 2–5) for PTTG1, and these counts were considered below the detection level that can be expected to provide reliable estimation of mRNA expression.

### DNA ploidy

We observed 111 (44%) patients with ≥1 tumor area with non-diploid DNA ploidy classification. DNA ploidy status was weakly correlated to the dichotomized scores of cytoplasmic BUB3 and CCNB1, but not to the PTTG1 scores (Supplementary Tables 3 and  4).

### Survival analyses

**Fig. 3:**
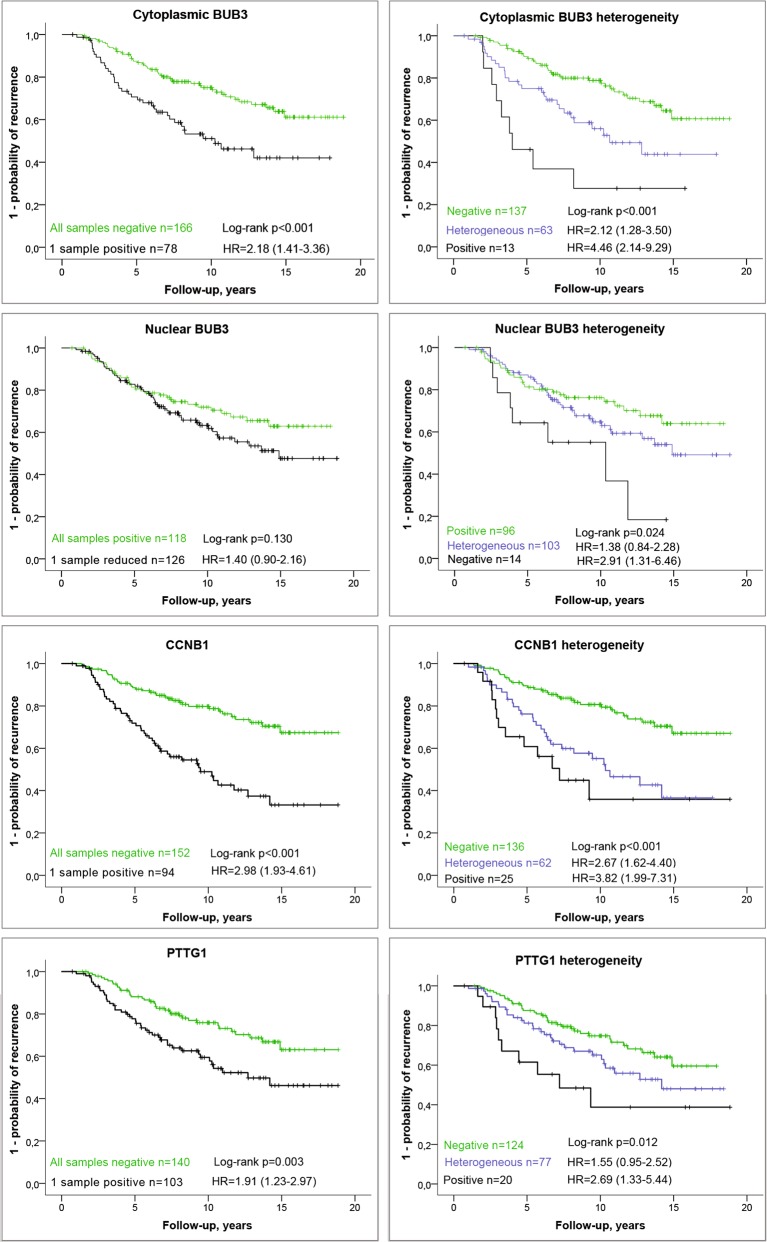
Univariable results of recurrence for cytoplasmic BUB3, nuclear BUB3, cyclin B1 (CCNB1), and pituitary tumor-transforming 1 (PTTG1). Left panel: A tumor was considered positive for cytoplasmic BUB3 (*n* = 244), decreased of nuclear BUB3 (*n* = 244), CCNB1 positive (*n* = 246), or PTTG1 positive (*n* = 243), if this score was observed in at least one of the three measured tumor areas. The threshold for a positve score was set to ≥5% positivity for cytoplasmic BUB3, 55 positive cells/mm^2^ for CCNB1 and 40 positive cells/mm^2^ for PTTG1. The threshold for decreased nuclear BUB3 was set to <99% positivity. Right panel: all patients with at least two valid results were included in analyses of heterogeneity of cytoplasmic BUB3 (*n* = 213), nuclear BUB3 (*n* = 213), CCNB1 (*n* = 223), or PTTG1 (*n* = 221). A tumor was considered heterogeneous when positive and negative tumor samples were observed in different tumor areas analyzed from the same tumor. Recurrence was defined as locoregional recurrence, metastasis or death from prostate cancer. The 95% confidence intervals of the hazard ratio (HR) are listed in parentheses.

Prognostic value was observed for the dichotomized scores of cytoplasmic BUB3, CCNB1, and PTTG1 in both univariable (Fig. 3) and multivariable analyses (Supplementary Table 5). DNA ploidy status was significant in univariable analysis only (hazard ratio [HR] = 2.33, 95% CI 1.51–3.60, *p* < 0.001), whereas nuclear BUB3 (Fig. 3) and BUB3 mRNA counts (Supplementary Fig. 2) were not significant.

### Combined risk assessment

**Fig. 4:**
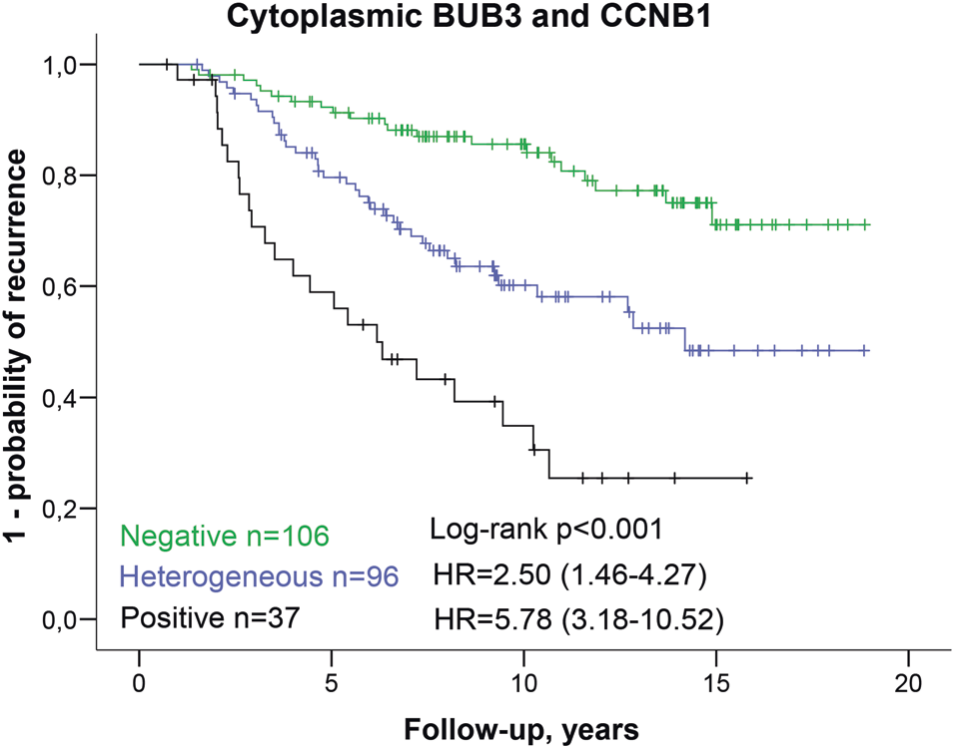
Univariable results of recurrence related to cytoplasmic BUB3 scores combined with cyclin B1 (CCNB1) scores. Valid results for both cytoplasmic BUB3 and CCNB1 were obtained for 239 patients. A tumor was considered positive or negative when a tumor was positive or negative for both of the proteins. Heterogeneous scores, where the tumors were positive for either cytoplasmic BUB3 or CCNB1, was observed in 96 patients. Recurrence was defined as locoregional recurrence, metastasis, or death from prostate cancer. The 95% confidence intervals of the hazard ratio (HR) are listed in parentheses.

When including cytoplasmic BUB3, CCNB1, and PTTG1 scores in a multivariable model with other clinicopathological variables, cytoplasmic BUB3 (HR = 2.29, 95% CI 1.39–3.80) and CCNB1 (HR = 2.28 95% CI 1.19–4.38) remained significant, while PTTG1 (HR = 1.08, 95% CI 0.56–2.10) did not (Supplementary Table 6). When combining the markers of cytoplasmic BUB3 and CCNB1, intermediate risk of recurrence was observed for patients with tumors positive for only one of the proteins (univariable HR = 2.50, 95% CI 1.46–4.27, multivariable HR = 2.55, 95% CI 1.44–4.53) and high risk for patients with tumors positive for both proteins (univariable HR = 5.78, 95% CI 3.18–10.52, multivariable HR = 4.25, 95% CI 2.11–8.53), compared with the low risk observed for patients with negative scores of both proteins (Fig. 4 and Table 2). CCNB1 and cytoplasmic BUB3 scores were integrated with the Cancer of the Prostate Risk assessment post-Surgical (CAPRA-S) score by adding two points for each marker if they were positive. The c-index of the modified CAPRA-S risk score was 0.78 (95% CI 0.73–0.83), compared with 0.76 (95% CI, 0.70–0.81) for the standard CAPRA-S. The observed difference of 0.02 between these c-indices was significant (95% CI 0.001–0.05, *p* = 0.044).

**Table 2:** Multivariable analysis (Cox-regression) of cytoplasmic BUB3 and cyclin B1 (CCNB1) scores combined.

Variable	HR	95% CI	*p* value
Cytoplasmic BUB3 & CCNB1			**<0.001**
Negative	Ref		
Heterogeneous	2.55	1.44–4.53	0.001
Positive	4.25	2.11–8.53	<0.001
Gleason score			**<0.001**
3 + 3	NA	NA	NA
3 + 4	Ref		
4 + 3	2.88	1.10–7.51	0.031
≥8	6.49	2.57–16.38	<0.001
Seminal vesicle invasion	1.52	0.91–2.53	0.108
Extracapsular extension	0.87	0.32–2.40	0.788
Lymph node invasion	1.99	0.90–4.39	0.090
Surgical margins	1.16	0.61–2.20	0.659
Preoperative PSA^a^			0.147^b^
≤6	Ref		
>6 and ≤10	1.19	0.39–3.63	0.764
>10 and ≤20	2.27	0.97–5.31	0.058
>20	2.28	0.96–5.38	0.061
Age^c^	0.96	0.92–0.99	**0.022**

*CI * confidence interval, *HR* Hazard ratio, *NA* not available

^a^ng/ml

^b^Preoperative PSA was a significant marker of recurrence when included as a continuous variable in a cox regression model: HR = 1.02, 95% CI 1.00–1.03, *p*  = 0.031

^c^Continuous variable

### Heterogeneity

A tumor, with at least two valid results, was considered heterogeneous when positive and negative scores were observed in different tumor areas from the same tumor. The percentage of patients with heterogeneous tumors (Table 3) was estimated for cytoplasmic BUB3 (30%), nuclear BUB3 (48%), CCNB1 (28%), and PTTG1 (35%). Patients with homogenous negative tumors of cytoplasmic BUB3, CCNB1 or PTTG1, or homogenous positive tumors of nuclear BUB3 were at lower risk of recurrence compared with patients with heterogeneous tumors. The highest risk of recurrence was observed for the patients with homogenous positive tumors of cytoplasmic BUB3, CCNB1 or PTTG1, or tumors with homogenous decreased expression of nuclear BUB3 (Fig. 3 and Table 3).

**Table 3:** Tumor heterogeneity.

	Block 1	Block 2	Block 3	Combined^a^
Cytoplasmic BUB3				
*n*	210	210	196	213
Negative	182	182	138	137
Heterogeneous	NA	NA	NA	63
Positive	28	28	58	13
*p* value^b^	0.089	**<0.001**	**0.001**	**<0.001**
HR (95% CI)	1.71 (0.91–3.21)	3.12 (1.83–5.30)	2.29 (1.41–3.73)	2.12 (1.28–3.50) 4.46 (2.14–9.29)
Nuclear BUB3				
*n*	210	210	196	213
Positive	157	141	139	96
Heterogeneous	NA	NA	NA	103
Reduced	53	69	57	14
*p* value^b^	0.518	**0.018**	0.152	**0.024**
HR (95% CI)	1.20 (0.70–2.06)	1.71 (1.09–2.68)	1.44 (0.87–2.40)	1.38 (0.84–2.28) 2.91 (1.31–6.46)
CCNB1				
*n*	221	207	214	223
Negative	152	157	173	136
Heterogeneous	NA	NA	NA	62
Positive	69	50	41	25
*p* value^b^	**<0.001**	**0.001**	**<0.001**	**<0.001**
HR (95% CI)	2.94 (1.86–4.63)	2.33 (1.42–3.82)	2.51 (1.51–4.19)	2.67 (1.62–4.40) 3.82 (1.99–7.31)
PTTG1				
*n*	214	201	209	221
Negative	149	152	167	124
Heterogeneous	NA	NA	NA	77
Positive	65	49	42	20
*p* value^b^	**0.036**	**0.041**	**0.014**	**0.012**
HR (95% CI)	1.65 (1.03–2.64)	1.68 (1.04–2.73)	1.95 (1.14–3.34)	1.55 (0.95–2.52) 2.69 (1.33–5.44)

*CI* confidence interval, *HR* hazard ratio, *NA* not available

^a^Patients were included in the combined analyses when a patient had ≥2 valid samples, a tumor was considered heterogeneous when both positive and negative scores were observed

^b^Log-rank *p* value

## Discussion

The present study is the largest published study on the prognostic value of BUB3, CCNB1, and PTTG1 expression in prostate cancer. We observed independent prognostic value of cytoplasmic BUB3 and CCNB1 when adjusted for PTTG1 and clinicopathological variables in multivariable analysis. Importantly, the highest risk of recurrence was observed for the combination of cytoplasmic BUB3 and CCNB1, which added moderately and significantly to the overall risk prediction, based on the CAPRA-S score.

BUB3 is constitutively expressed and normally restricted to the nucleus [16] where it acts as a mitotic checkpoint protein. Correspondingly, we observed that most interphase cells stained positive for nuclear BUB3. In our study, counts of mRNA was weakly associated with protein levels of nuclear BUB3, neither were associated with recurrence. The lack of significant results may be explained by the fact that only one block was included in the analysis of mRNA, as significant results were obtained for nuclear BUB3 in the analysis that considered intra-tumor heterogeneity. This analysis included two or three tumor samples from each patient and demonstrated that patients with decreased levels of nuclear BUB3 in all analyzed samples were at increased risk of recurrence. Zhu and colleagues [16] identified a nuclear localization signal in the amino acid sequence of BUB3 in HeLa cells. A mutation in the nuclear localization signal of BUB3 resulted in both cytoplasmic retention and nuclear expression and reduced the ability of BUB3 to arrest cells in mitosis [16]. We observed cytoplasmic expression of BUB3 in 32% of the cases and the presence of BUB3 in the cytoplasm indicated disease recurrence. The BUB3 pre-absorbed with blocking peptides was non-reactive in the tested samples, attesting to the specificity and validity of the observed cytoplasmic staining.

CCNB1 and PTTG1 expression is restricted to cells undergoing cell division [17, 18]. The low expression of CCNB1 and PTTG1 observed in this study is in agreement with the low proliferation rates in prostate cancer [19] and comparable to the protein levels found in small-scale studies in prostate cancer [20, 21]. The expression pattern of CCNB1 and PTTG1 is expected to be correlated as the degradation of both proteins is regulated by the mitotic checkpoint and completed before the mitotic exit [17, 18]. In our study, the categorized expression of the two genes was significantly correlated with equivalent scores in 81% of the tumors. However, we observed a higher median expression of CCNB1 compared with PTTG1 which contradicts the reported longer half-lives of PTTG1 when compared with CCNB1 [22]. The overexpression of CCNB1 may be contributed to either unscheduled expression of CCNB1 in G_1_ phase [23] or sustained expression of CCNB1 as a consequence of G_2_ arrest [24]. Upregulation of PTTG1 was shown in a study describing a gene-expression signature connected to metastasis in solid tumors, including prostate cancer [25], and underscores the potential usefulness of this gene in prostate cancer prognostication. However, only CCNB1 remained significant when both proteins were included in a multivariable analysis.

Deregulation of genes involved in the mitotic checkpoint, such as BUB3 [26], CCNB1 [27], and PTTG1 [28], can contribute to cancer development by increasing the risk of incorrect cell division, aneuploidy, and genomic instability [29]. We observed that DNA ploidy was weakly associated to cytoplasmic BUB3 and CCNB1 expression. As multiple genes and processes contribute to genomic instability [30], an association should not necessarily be expected when only a few of them are studied, particularly in samples collected at a single time-point given by surgery date. Furthermore, studies in cell lines suggests that cancer cells depend on a functional mitotic checkpoint [4] and increased expression of CCNB1 and PTTG1 is therefore more likely a result of increased proliferation. This may explain why non-diploid tumors were equally distributed in tumors with positive and negative cytoplasmic BUB3 and CCNB1 expression.

Scoring of PTTG1 and CCNB1 may prove challenging due to the low abundance of these proteins in prostate cancer. An automatic scoring system was used to overcome this challenge, allowing us to count positive tumor cells in an accurate and reproducible manner, thereby eliminating observer variability. The expression of BUB3 was only scored visually as the automatic scoring could not be applied due to technical challenges. However, the inter-observer agreement was substantial. Another limitation included the use of a patient cohort from the pre-PSA era containing more aggressive tumors compared with contemporary cohorts. As a consequence, the relevance of these markers should be validated in a cohort that is more representative for patients diagnosed today, including biopsy material from patients under active surveillance. However, the long follow-up gave us the advantage of using clinical recurrence as an endpoint in the survival analyses. A sampling bias caused by limited tumor material for some of the patients, may have excluded patients with a low tumor burden. Furthermore, some tumor samples were excluded as the tumor tissue fell off during the immunohistochemistry procedure. This is a well-known issue [31], which we tried to avoid by using coated slides and removing excess water in the sections before freezing.

Intra-tumor heterogeneity is a challenge for biomarker studies. The heterogeneity of gene expression of BUB3, CCNB1, and PTTG1 was assessed at the protein level, and observed in about one third of the tumors. The prognostic value improved when we combined the results from three tissue blocks from a patient into one score for each of the proteins. This is in line with the previously described impact of heterogeneity on DNA ploidy as a prognostic marker [1]. Our results indicate that it is possible to account for intra-tumor heterogeneity by analysis of multiple tumor samples, which is a disadvantage in a clinical setting. On the other hand, univariable analyzes of the separate series of tumor samples were statistically significant, which means that one tissue block per patient may be sufficient to assess CCNB1 and PTTG1 expression. Significant results were only obtained for two of the series for cytoplasmic BUB3 and one series for nuclear BUB3, which necessitates analyses of multiple tumor samples for this protein. However, our three-tier system indicates that the presence of intra-tumor heterogeneity is a marker of prognostic importance in itself. We would therefore recommend that the evaluation of these proteins should be examined in more than one tissue block for each patient.

## Conclusion

The prognostic value of cytoplasmic BUB3 expression in prostate carcinomas has been reported for the first time in this study. The positive expression of cytoplasmic BUB3, CCNB1, and PTTG1 was significantly correlated with recurrence. Evaluation of the investigated proteins in three tissue blocks considerably improved their prognostic value. The combination of cytoplasmic BUB3 and CCNB1 stratified the patients into three risk groups with a 10-year recurrence rate of 16% for the low-risk group, 40% for the intermediate risk group and 65% for the high-risk group. This marker has the potential to enable better risk stratification of patients with prostate cancer, as it significantly improved the CAPRA-S risk stratification tool.

## Supplementary information

Supplemental material
