# Supplementary material for: Prognostic value of mitotic checkpoint protein BUB3, cyclin B1, and pituitary tumor-transforming 1 expression in prostate cancer
Source: Mod Pathol. 2019 Dec 4;33(5):905–15. doi: 10.1038/s41379-019-0418-2 (PMC7190565; doi:10.1038/s41379-019-0418-2)
Supplement: Supplementary file 1 — Supplemental material [file 41379_2019_418_MOESM1_ESM.docx]

**Supplemenatry methods**

Extraction and quantification of RNA: The 297 separate tumor areas were dissected from one tissue block from the 253 patients. RNA was extracted using RecoverAll™ Total Nucleic Acid Isolation Kit (Ambion, Austin, TX). Fifty to 100 ng of RNA from each sample was analyzed by multiplex digital color-coded barcode hybridization technology (NanoString Technologies™, Seattle, WA). Probe sets, targeting all isoforms of both BUB3 and CCNB1, and two of three isoforms of PTTG1, were designed and synthesized by NanoString nCounter™ technologies. The raw probe counts were normalized in nSolver™ v3.0 to the geometric mean of the positive controls and the geometric mean of five housekeeping genes with a CV<65% (*G6PD*, *LDHA*, *PGK1*, *RPL19* and *TUBB*).

**Supplemenatry Figures and Tables**

**
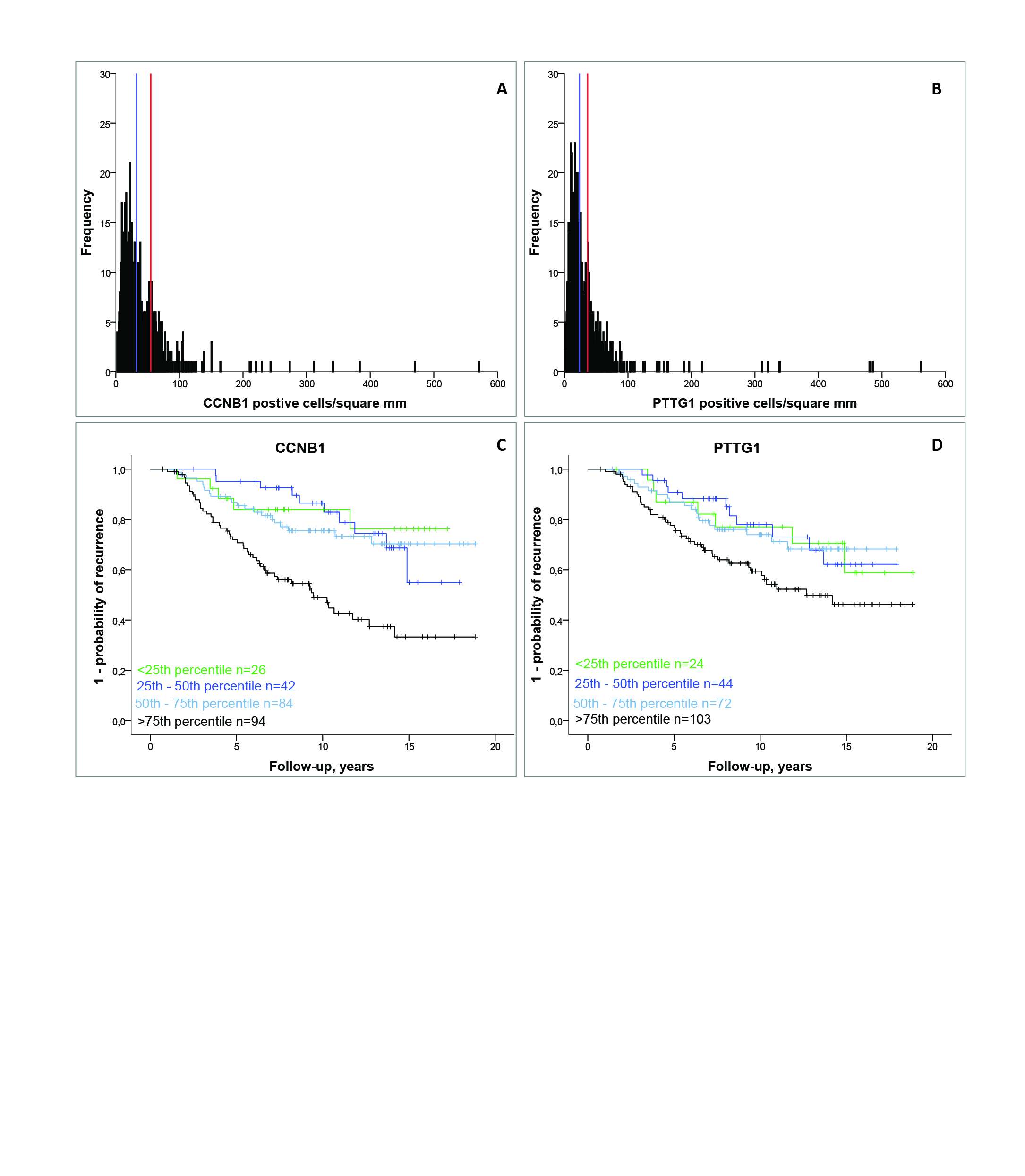
**

**Supplementary Figure 1: Threshold for dichotomizing the automatic scores of cyclin B1 (CCNB1) and pituitary tumor-transforming 1 (PTTG1).** The number of postive cells in a tumor area was scored automatically, and positive cells/mm^2^ was calculated. **(A)** The distribution of automatic scores of CCNB1. A median of 31 (IQR 18-55) positive cells/mm^2^ was observed for the 642 samples scored for CCNB1. **(B)** The distribution of automatic scores of PTTG1. A median of 24 (IQR 14-40) positive cells/mm^2^ was observed for the 624 samples scored for PTTG1**.** The median is marked with a blue line and the 75^th^ percentile with a red line. The left-skewed distribution of the automatic scores of CCNB1 and PTTG1 suggested that the 75^th^ percentile could be considered a reasonable cut-off for increased protein levels. This was confirmed by the survival curves for both **(C)** CCNB1 (n=246) and **(D)** PTTG1 (n=243), when the data were categorized into four groups by the 25^th^, 50^th^ and 75^th^ percentiles. A tumor was considered positive when at least one of the samples was positive. The three groups with the lowest protein levels clustered together in the survival plot and we dichotomized the automatic scores by the 75^th^ percentile in the analyses.


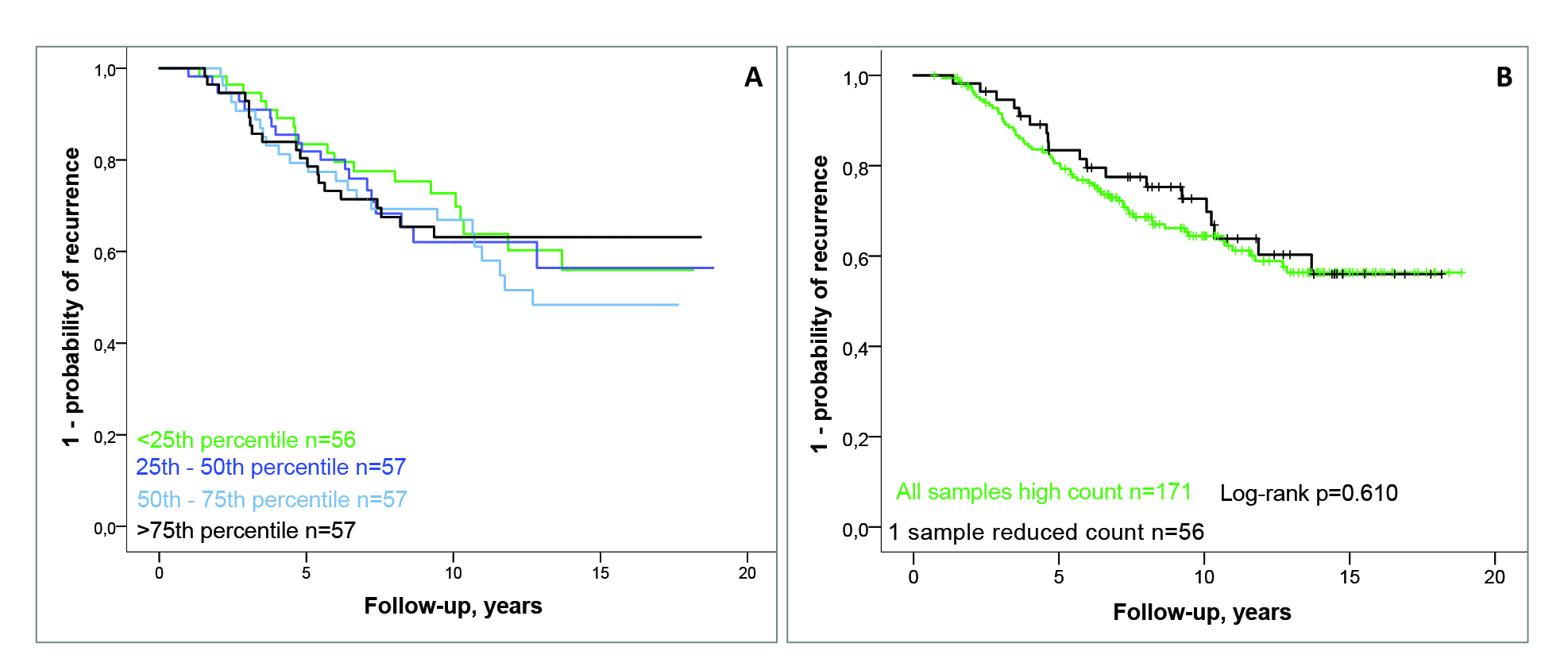


**Supplementary Figure 2: Threshold for dichotimizing mitotic checkpoint protien BUB3 mRNA counts and univariable results.** Counts of BUB3 mRNA (n=227) were analyzed in tumor material from block 1. **(A)** The counts were categorized by the 25th (52 mRNA transcripts), 50th (67 mRNA transcripts) and 75th (81 transcripts) percentiles. Time to recurrence was similar in all four groups. **(B)** We dichotomized the BUB3 mRNA count by the 25th percentile in the following analyses, as decreased levels of nuclear BUB3 protein was observed for approximately 25% of the patients in the corresponding tissues section investigated by immunohistochemistry. Decreased count of BUB3 mRNA was observed in 56 patients. There was no significant difference in time to recurrence for patients with different mRNA levels for BUB3 in univariable analysis (p=0.610).

| **Supplementary Table 1: Overview of antibodies used for immunohistochemistry** | | | |
| --- | --- | --- | --- |
| Antibody | Clone | Dilution | Supplier |
| **BUB3^a^**  Rabbit anti-Human monoclonal (N-terminus) | EPR5319(2) | 1:4000 | LSBio  Seattle, WA |
| **BUB3 ^a^**  Rabbit anti-Human monoclonal (N-terminus) | EPR5319(2) | 1:2000 | Abcam  Cambridge, UK |
| **CCNB1**  Rabbit anti-human monoclonal | Y106 | 1:200 | Abcam  Cambridge, UK |
| **PTTG1**  Mouse anti-human monoclonal | DCS-280 | 1:600 | LSBio  Seattle, WA |
| **^a^**Tissue sections from block 1 and 2 were incubated with BUB3 antibody from LSBio. As the product was no longer available from LSBio, sections from block 3 were incubated with the same BUB3 antibody clone, from a different supplier.  Abbreviations: BUB3=mitotic checkpoint protein BUB3, CCNB1=cyclin B1, PTTG1=pituitary tumor-transforming 1 | | | |

| Supplementary Table 2: Settings for automatic scoring of immune by ImmunoPath | | |
| --- | --- | --- |
|  | CCNB1 | PTTG1 |
| Protocol | Count | Count |
| Positive  H (min/max)  S (min/max)  V (min/max) | 0.89/0.15  0.11/0.96  0.07/0.79 | 0.98/0.10  0.15/0.93  0.17/0.83 |
| Preprocessor commands | Median_3x3  HoleFill | Median_3x3  HoleFill |
| BlobFilter | Area >100  Area <2000 | Area >100  Area <2000 |
| Abbreviations: CCNB1=cyclin B1, PTTG1=pituitary tumor-transforming 1 | | |

| **Supplementary Table 3: Correlation between mitotic checkpoint protein BUB3 and clinical variables** | | | | | | |
| --- | --- | --- | --- | --- | --- | --- |
|  | **Protein** | | | | **mRNA^a^** | |
| **Variable** | **Nuclear BUB3** | | **Cytoplasmic BUB3** | | **BUB3** | |
|  | **CC** | **p-value** | **CC** | **p-value** | **CC** | **p-value** |
| Nuclear BUB3 | 1 |  | 0.22 | **<0.001** | 0.16 | **0.027** |
| Cytoplasmic BUB3 | 0.22 | **<0.001** | 1 |  | -0.13 | 0.072 |
| PSA^b^ | 0.08 | 0.159 | -0.02 | 0.691 | 0.04 | 0.565 |
| Gleason score^c^ | 0.07 | 0.241 | 0.14 | **0.018** | 0.04 | 0.493 |
| Surgical margins | 0.08 | 0.190 | 0.11 | 0.076 | 0.04 | 0.526 |
| Seminal vesicle invasion | -0.02 | 0.761 | 0.21 | **0.001** | -0.03 | 0.656 |
| Extracapsular extension | -0.02 | 0.785 | 0.16 | **0.014** | -0.001 | 0.985 |
| Lymph node metastases | -0.04 | 0.500 | -0.06 | 0.386 | 0.02 | 0.728 |
| DNA ploidy^d^ | 0.09 | 0.179 | 0.15 | **0.019** | 0.04 | 0.570 |
| Associations were evaluated using the Pearson’s χ^2^ test for categorical variables and Kendall’s τ test for ordinal variables.  ^a^Correlation between mRNA and protein is performed on the single block of mRNA and the immune sections from the corresponding tumor blocks. ^b^Preoperative PSA (ng/ml) ordinal (≤6, >6 and ≤10, >10 and ≤20, >20). ^c^Ordinal (≤6, 3+4, 4+3, ≥8). ^d^Categorical (diploid, non-diploid).  Abbreviations: CC=correlation coefficient. | | | | | | |

| **Supplementary Table 4: Correlation between cyclin B1 (CCNB1),**  **pituitary tumor-transforming 1 (PTTG1) and clinical variables** | | | | | | | | |
| --- | --- | --- | --- | --- | --- | --- | --- | --- |
|  | **CCNB1** | | | | **PTTG1** | | | |
| **Variable** | **Automatic** | | **Visual** | | **Automatic** | | **Visual** | |
|  | **CC** | **p-value** | **CC** | **p-value** | **CC** | **p-value** | **CC** | **p-value** |
| Visual | 0.58 | **<0.001** | 1 |  | 0.46 | **<0.001** | 1 |  |
| PSA^a^ | 0.01 | 0.823 | -0.02 | 0.705 | -0.05 | 0.382 | -0.01 | 0.819 |
| Gleason score^b^ | 0.28 | **<0.001** | 0.26 | **<0.001** | 0.15 | **0.010** | 0.14 | **0.017** |
| Surgical margins | 0.06 | 0.358 | 0.09 | 0.178 | 0.05 | 0.465 | 0.04 | 0.569 |
| Seminal vesicle invasion | 0.26 | **<0.001** | 0.19 | **0.003** | 0.19 | **0.004** | 0.14 | **0.028** |
| Extracapsular extension | 0.20 | **0.002** | 0.18 | **0.006** | 0.03 | 0.693 | 0.16 | **0.015** |
| Lymph node metastases | 0.04 | 0.547 | 0.01 | 0.846 | 0.002 | 0.971 | -0.03 | 0.675 |
| DNA ploidy^c^ | 0.18 | **0.005** | 0.13 | **0.047** | 0.07 | 0.306 | 0.09 | 0.180 |
| Associations were evaluated using the Pearson’s χ^2^ test for categorical variables and Kendall’s τ test for ordinal variables.  ^a^Preoperative PSA (ng/ml) ordinal (≤6, >6 and ≤10, >10 and ≤20, >20). ^b^Ordinal (≤6, 3+4, 4+3, ≥8). ^c^Categorical (Diploid, non-diploid).  Abbreviations: CC=correlation coefficient. | | | | | | | | |

| **Supplementary Table 5: Multivariable analyses including the dichotomized protein levels and clinicopathological variables** | | | | | | | | | |
| --- | --- | --- | --- | --- | --- | --- | --- | --- | --- |
|  | **Cytoplasmic BUB3** | | | **CCNB1** | | | **PTTG1** | | |
| **Variable** | **HR** | **95% CI** | **p-value** | **HR** | **95% CI** | **p-value** | **HR** | **95% CI** | **p-value** |
| Protein | 2.02 | 1.25-3.27 | **0.004** | 2.27 | 1.40-3.69 | **0.001** | 1.87 | 1.13-3.10 | **0.015** |
| Gleason score |  |  | **<0.001** |  |  | **<0.001** |  |  | **<0.001** |
| ≤6 | NA | NA | NA | NA | NA | NA | NA | NA | NA |
| 3+4 | Ref |  |  | Ref |  |  | Ref |  |  |
| 4+3 | 2.24 | 0.94-5.31 | 0.068 | 2.96 | 1.16-7.54 | 0.023 | 2.11 | 0.88-5.07 | 0.094 |
| ≥8 | 5.28 | 2.29-12.13 | <0.001 | 6.91 | 2.77-17.25 | <0.001 | 4.84 | 2.09-11.19 | <0.001 |
| Seminal vesicle invasion | 1.73 | 1.06-2.83 | **0.029** | 1.66 | 1.02-2.72 | **0.042** | 1.67 | 1.01-2.77 | **0.045** |
| Extracapsular extension | 1.11 | 0.41-2.99 | 0.841 | 1.04 | 0.38-2.86 | 0.945 | 1.27 | 0.46-3.46 | 0.644 |
| Lymph node metastases | 1.58 | 0.72-3.48 | 0.255 | 1.48 | 0.68-3.24 | 0.324 | 1.63 | 0.74-3.60 | 0.224 |
| Surgical margins | 1.31 | 0.71-2.45 | 0.389 | 1.27 | 0.71-2.28 | 0.417 | 1.48 | 0.81-2.71 | 0.204 |
| Preoperative PSA**^a^** |  |  | 0.241**^b^** |  |  | 0.266**^c^** |  |  | 0.193**^d^** |
| ≤6 | Ref |  |  | Ref |  |  | Ref |  |  |
| >6 and ≤10 | 1.21 | 0.40-3.67 | 0.742 | 1.05 | 0.36-3.07 | 0.929 | 1.22 | 0.40-3.71 | 0.733 |
| >10 and ≤20 | 2.12 | 0.93-4.81 | 0.073 | 1.89 | 0.82-4.31 | 0.133 | 2.18 | 0.96-4.95 | 0.063 |
| >20 | 1.90 | 0.82-4.40 | 0.135 | 1.89 | 0.81-4.41 | 0.138 | 2.10 | 0.89-4.95 | 0.089 |
| Age**^e^** | 0.97 | 0.93-1.01 | 0.095 | 0.96 | 0.92-0.99 | **0.024** | 0.96 | 0.92-0.99 | **0.025** |
| ^a^ng/ml. ^b^Preoperative PSA was not significant when included as a continuous variable in a cox regression model: HR=1.01, 95% CI 0.99-1.02, p=0.104. ^c^Preoperative PSA was a significant marker of recurrence when included as a continuous variable in a cox regression model: HR=1.02, 95% CI 1.00-1.03, p=0.027. ^d^Preoperative PSA was not significant when included as a continuous variable in a cox regression model: HR=1.01, 95% CI 0.99-1.03, p=0.075. ^e^Continuous variable. | | | | | | | | | |
| Abbreviations: CCNB1=cyclin B1, CI=confidence interval, HR=hazard ratio NA=not available, PTTG1=pituitary tumor-transforming 1, Ref=reference group. | | | | | | | | | |

| **Supplementary Table 6: Multivariable analysis of cytoplasmic BUB3, cyclin B1 (CCNB1) and pituitary tumor-transforming 1** **(PTTG1) scores** | | | |
| --- | --- | --- | --- |
| **Variable** | **HR** | **95% CI** | **p-value** |
| Cytoplasmic BUB3 | 2.29 | 1.39-3.80 | **0.001** |
| CCNB1 | 2.28 | 1.19-4.38 | **0.013** |
| PTTG1 | 1.08 | 0.56-2.10 | 0.816 |
| Gleason score |  |  | **<0.001** |
| ≤6 | NA | NA | NA |
| 3+4 | Ref |  |  |
| 4+3 | 3.20 | 1.23-8.34 | 0.017 |
| ≥8 | 6.77 | 2.66-17.20 | <0.001 |
| Seminal vesicle invasion | 1.38 | 0.82-2.31 | 0.227 |
| Extracapsular extension | 0.82 | 0.30-2.27 | 0.701 |
| Lymph node metastases | 2.44 | 1.10-5.44 | **0.029** |
| Surgical margins | 1.03 | 0.53-1.98 | 0.935 |
| Preoperative PSA^a^ |  |  | 0.199^b^ |
| ≤6 | Ref |  |  |
| >6 and ≤10 | 1.24 | 0.40-3.85 | 0.712 |
| >10 and ≤20 | 2.16 | 0.93-5.03 | 0.075 |
| >20 | 2.28 | 0.93-5.60 | 0.072 |
| Age^c^ | 0.95 | 0.91-0.99 | **0.025** |
| ^a^ng/ml. ^b^Preoperative PSA was not significant when included as a continuous variable in a cox regression model: HR=1.01, 95% CI 0.99-1.03, p=0.125. ^c^Continuous variable. | | | |
| Abbreviations: CI=confidence interval, HR=hazard ratio, NA=not available. | | | |
